# Diabetes, lowered mental health functioning and the use of conventional and complementary medicine: results from a secondary analysis of the complementary medicine use, health literacy and disclosure (CAMUHLD) study

**DOI:** 10.1186/s12906-025-04876-0

**Published:** 2025-04-16

**Authors:** Tracey Oorschot, Jon Adams, David Sibbritt

**Affiliations:** School of Public Health, University of Technology Sydney, Broadway, PO Box 123, Sydney, NSW 2007 Australia

**Keywords:** Integrative medicine, Complementary medicine, Chronic disease, Diabetes, Mental health

## Abstract

**Background:**

Diabetes Mellitus is often a long-term health condition that continues to raise concerns regarding the burden upon an individual’s mental health, due to the commitment required for day-to-day self-care. People living with diabetes frequently use complementary medicine as part of their diabetes self-care to manage their mental health and this raises a number of significant risk management issues. Unfortunately, no research has explored the influence of lowered mental health functioning upon both the conventional and complementary medicine health service use amongst people living with diabetes.

**Methods:**

An examination of the conventional and complementary medicine health service use amongst men and women living with diabetes and normative or lowered mental health functioning, was undertaken by completing a secondary analysis of the Complementary Medicine Use, Health Literacy and Disclosure study.

**Results:**

Of the 176 participants reporting a diabetes diagnosis, 74% reported lowered mental health functioning, compared to 60% without a diabetes diagnosis. Compared to people living with diabetes and normative mental health functioning, those with lowered mental health functioning were 9 times more likely to consult with a Western herbalist (OR = 9.17, 95% CI: 1.097–76.84), twice as likely to use vitamins or minerals (OR = 2.34, 95% CI: 1.061–5.151), and 5 times more likely to engage in relaxation or meditation practice (OR = 5.10, 95% CI: 1.362–19.129).

**Conclusion:**

People living with diabetes who have lowered mental health functioning appear even more likely to use complementary medicine than conventional medicine, than those with normative mental health functioning. This reinforces the need to resolve clinical governance issues associated with complementary medicine use, especially what role complementary medicine practitioners can fulfil as part of coordinated diabetes care teams, to support patient health and well-being.

## Background

Diabetes Mellitus is often a long term health condition with significant impacts upon an individual’s physical and mental health [1, 2]. People living with diabetes are known to experience elevated levels of psychological distress [3, 4, 5, 6]. Psychological distress reflects lowered mental health functioning such as experiencing general anxiety (e.g. nervousness), lowered mood (e.g. sadness) or stress (e.g. loss of sense of calm) [4, 7]. For people living with diabetes, psychological distress correlates more closely with diabetes distress - an emotional response that people living with diabetes can experience due to the challenges in the ongoing commitment of day-to-day diabetes self-management [8, 9], rather than clinical levels of anxiety or depressive disorders [8, 10]. Whilst diabetes distress affects around one third of people living with diabetes, it remains a neglected aspect of diabetes research, with leading diabetes psychosocial researchers highlighting the urgent need for more data regarding the psychological aspects of diabetes [9]. Furthermore, psychological distress often adversely impacts diabetes self-care behaviours such as adherence to a healthy diet, exercise and blood glucose monitoring [4, 5]. Consequently, managing psychological health becomes an important aspect of diabetes self-care.

People living with diabetes also frequently use complementary medicine (CM) as part of their diabetes self-care to manage their mental health [6, 11]. CM includes practices (e.g. yoga or meditation), products (e.g. herbal medicines) or consultation with practitioners (e.g. Western Herbalist) [12]. CM use has been associated with the presence of lowered mental health functioning [13], as well for those wanting to improve or maintain their health and wellbeing [14]. CM use patterns in association with lowered mental health functioning and CM practitioner groups, products and practices has limited research [15]. Previous research reveals practice use of relaxation techniques and massage, and product use including herbal medicines [16, 17]. Commonly consulted practitioners who screen for mental health concerns include naturopaths and herbalists [18, 19].

CM use by those living with diabetes raises a number of significant risk management issues [20] especially with regards to contraindications between CM products and commonly used diabetes therapies [21, 22]– a risk exacerbated by the high rates of non-disclosure by CM users to conventional medical providers [23, 24].

More recently, a small but growing body of clinical research has begun to consider coordinated care approaches that can better support the specific needs of people living with diabetes [25, 26, 27] or mental health concerns [28, 29, 30] who choose to use CM as part of their self-care. Such coordinated approaches to clinical diabetes care have been further supported by broader care models (beyond diabetes care) including collaboration between conventional and CM practitioners which appear to result in better health outcomes for people with physical and mental health co-morbidities [31, 32].

Despite increasing interest in improving self-care support for people living with diabetes [21, 33], and the known negative impacts of poor mental health upon diabetes self-care, no research to date has explored the influence of lowered mental health functioning upon the health service use amongst people living with diabetes. This study addresses this research gap by exploring *both* conventional and CM health service use of men and women living with diabetes and normative or lowered mental health functioning.

## Methods

### Study design

The results reported in this paper are derived from a secondary analysis of the Complementary Medicine Use, Health Literacy and Disclosure (CAMUHLD) study. CAMUHLD participants were asked a series of questions regarding socio-demographic characteristics, health status and health service use. Ethics approval was obtained from the Endeavour College of Natural Health, Charles Sturt University, the University of Sydney and the University of Technology, Sydney.

### Study population

The CAMUHLD study was a nationally representative [34] cross-sectional survey of Australian residents aged 18 years and over, with the data collected in 2017. A total of 41,000 people were emailed with an invitation to complete the online questionnaire. A total of 2,019 men and women completed the questionnaire.

### Study sample

Participants who reported a diabetes diagnosis (*n* = 176) of either type 1 or 2 diabetes mellitus were selected for further analysis. Respondents were grouped by mental health status and classified as either with lowered or normative mental health functioning.

### Mental health status

The CAMUHLD study included the Short form Health Survey (SF-20) that assesses health quality of life across six different dimensions of health, with one dimension relating to mental health (MHI-5 subscale) [35]. The MH1-5 scale comprises five items measuring general mental health symptoms in the previous month using a 6-point scale (1 = all of the time to 6 = none of the time). Higher scores correlate with increasing mental health functioning, with a cut-off point of a score of 73 or more indicative of normative functioning, whereas a score of 72 or below indicates lowered mental health functioning [36]. Participants with a score of 73 or more were classified as (no) in relation to lowered functioning (mental health), with those with a score of 72 or less classified as (yes) in relation to lowered functioning (mental health).

### Health service use

The CAMUHLD survey questioned participants about their conventional and CM health service use over the previous 12 months. This included consultation with conventional medicine practitioners such as a general practitioner, allied health worker (e.g. psychologist), hospital doctor or medical specialist, and use of conventional medicine such as over-the-counter or prescription medicine products. The survey also questioned participants about their CM health care use including CM practitioner consultations, their use of CM products including herbal medicines or vitamins/minerals and use of CM practices such as yoga or relaxation methods.

Consultation with either a counsellor or psychologist were grouped together in the one category, as were dietitian or nutritionist. Practice use regarding yoga or tai chi, and relaxation or meditation practices were also combined. Frequency of consultation with a general practitioner were categorised into low (1–2 visits), medium (3–6 visits) or high (7 or more visits) annually.

### Statistical analyses

The statistical software package Stata, version 16.1 was used to complete all of the statistical analyses. To determine if any significant associations existed between mental health functioning and a diabetes diagnosis, bivariate analyses were completed, using chi-squared tests or Fisher’s exact test, where appropriate. Similarly, to determine bivariate associations between mental health status and socio-demographic, health status and health service use variables were assessed using chi-squared tests or Fisher’s exact test, where appropriate. Following the bivariate analyses, all socio-demographic, health status and health service use variables that had a bivariate p-value < 0.25 [37] were entered into a model and then a stepwise backward elimination process was employed, using a likelihood ratio test, to eventually produce the most parsimonious model. Statistical significance was set at a p-value of < 0.05.

## Results

There was a significant association (*p* < 0.05) between mental health functioning and a diabetes diagnosis (see Table 1). Of the people reporting a diabetes diagnosis, 26.1% (*n* = 46) were classified with normative mental health functioning, and 73.9% (*n* = 130) with lowered mental health functioning.

**Table 1:** Association of mental health status of participants with and without diabetes mellitus

Mental Health Status	Diabetes Status		
	No (*n* = 1,843)	Yes (*n* = 176)	p-value
***Lowered functioning***			< 0.001
No	737 (40.00)	46 (26.14)	
Yes	1106 (60.00)	130 (73.86)	

### Socio-demographic and health status characteristics

Table 2 reports the socio-demographic and health status characteristics of people with diabetes by mental health status. Age, employment status, and financial situation were significantly (*p* < 0.05) associated with mental health functioning. People aged 60 and over, not employed and experiencing some financial difficulty had the highest proportions of lowered mental health functioning.

**Table 2:** Socio-demographic and health status characteristics of people with diabetes mellitus by mental health status

Characteristic			
	Mental Health Status (lowered functioning) **(** ***n*** ** = 176)**		
	No **(** ***n*** ** = 46)**	Yes **(** ***n*** ** = 130)**	*p*-value
	n (%)	n (%)	
**Socio-demographic**			
**Gender**			
Male	33 (71.7)	73 (56.2)	0.063
Female	13 (28.3)	57 (43.8)	
**Age**			0.014
18–29	3 (6.5)	27 (20.8)	
30–39	5 (10.9)	11 (8.5)	
40–49	4 (8.7)	19 (14.6)	
50–59	5 (10.9)	26 (20.0)	
60+	29 (63.0)	47 (36.1)	
**Area of residence**			
Major cities	32 (69.6)	92 (70.8)	0.447
Inner regional	12 (26.1)	26 (20.0)	
Outer reg/remote	2 (4.3)	12 (9.2)	
**Marital Status**			
Single	10 (21.7)	23 (17.7)	0.747
Married/de facto	28 (60.9)	79 (60.8)	
Separated/divorced/widowed	8 (17.4)	28 (21.5)	
**Employment Status**			0.005
Fulltime	5 (10.9)	43 (33.1)	
Parttime/casual	6 (13.0)	22 (16.9)	
Not employed	35 (76.1)	65 (50.0)	
**Highest Qualification**			
No school/school/high school cert	14 (30.4)	38 (29.2)	0.475
Trade/Tertiary cert/diploma	21 (45.6)	49 (37.7)	
University degree	11 (23.9)	43 (33.1)	
**Financial situation**			
No or little difficulties	19 (41.3)	35 (26.9)	0.024
Some difficulties	21 (45.6)	52 (40.0)	
Struggles with income	6 (13.0)	43 (33.1)	
**Health care card status**			
No	27 (58.7)	91 (70.0)	0.161
Yes	19 (41.3)	39 (30.0)	
**PHI status** ^**a**^			
No	23 (50.0)	68 (52.3)	0.788
Yes	23 (50.0)	62 (47.7)	
**Health Status**			
**General Health**			
Poor/fair/good	32 (69.6)	99 (76.1)	0.379
Very good/excellent	14 (30.4)	31 (23.9)	
**Heart Disease**			
No	41 (89.1)	110 (84.6)	0.451
Yes	5 (10.9)	20 (15.4)	
**Hypertension**			
No	29 (63.1)	82 (63.1)	0.997
Yes	17 (36.9)	48 (36.9)	

Notes:

a: health care card = eligibility for concessional health services within Australia

b: PHI = private health insurance coverage for hospital or general treatment services (e.g. physiotherapy) by a private health care provider

### Prevalence of conventional and complementary medicine health service use

From Table 3, health service use was significantly (*p* < 0.05) associated with consultation with a counsellor or psychologist, podiatrist, chiropractor and Western Herbalist. Those with lowered mental health functioning had less tendency to visit a specialist doctor or podiatrist, but more likely to visit a counsellor or psychologist, chiropractor or Western Herbalist. Use of aromatherapy oils, herbal medicines, yoga or tai chi, relaxation or meditation, vitamin/minerals and flower essences were also significantly (*p* < 0.05) associated with mental health functioning. People with lowered mental health functioning showed a higher proportion of aromatherapy oil, herbal medicine, yoga or tai chi, relaxation or meditation, vitamin/minerals and flower essence use compared to those with normative mental health functioning.

**Table 3:** Prevalence of health service use of people with diabetes mellitus by mental health status

Health Service Use (yes/no)		Mental Health Status (lowered functioning)		
		**(** ***n*** ** = 176)**		
		No **(** ***n*** ** = 46)**	Yes **(** ***n*** ** = 130)**	*p*-value
		**n (%)**	**n (%)**	
**Practitioners**				
*Conventional*				
***GP***	Low 1–2	11 (23.9)	25 (21.2)	*0.574*
	Med 3–6	20 (43.5)	44 (37.3)	
	High 7+	15 (32.6)	49 (41.5)	
***Specialist doctor*** no yes		12 (26.1)	52 (40.0)	*0.092*
		34 (73.9)	78 (60.0)	
***Hospital doctor*** no Yes		22 (47.8)	61 (46.9)	*0.916*
		24 (52.2)	69 (53.1)	
***Nurse*** no yes		33 (71.7)	98 (75.4)	*0.626*
		13 (28.3)	32 (24.6)	
***Pharmacist*** no yes		3 (6.5)	20 (15.4)	*0.125*
		43 (93.5)	110 (84.6)	
***Counsellor/psychologist*** no yes		39 (84.8)	80 (61.5)	*0.004*
		7 (15.2)	50 (38.5)	
***Podiatrist*** no		44 (95.6)	130 (100)	*0.017*
yes		2 (4.3)	0 (0)	
***Exercise physiologist*** no		45 (97.9)	129 (99.2)	*0.440*
yes		1 (2.2)	1 (0.8)	
***Physiotherapist*** no yes		31 (67.4)	90 (69.2)	*0.817*
		15 (31.6)	40 (30.8)	
***Dietitian/nutritionist*** no yes		46 (100)	129 (99.2)	*1.000*
		0 (0)	1 (0.8)	
*Complementary Medicine*				
***Acupuncturist*** no yes		44 (95.6)	111 (85.4)	*0.065*
		2 (4.35)	19 (14.6)	
***Chiropractor*** no yes		43 (93.5)	105 (80.8)	*0.043*
		3 (6.5)	25 (19.2)	
***Naturopath*** no yes		41 (89.1)	114 (87.7)	*0.796*
		5 (10.9)	16 (12.3)	
***Western herbalist*** no		45 (97.8)	112 (86.1)	*0.028*
yes		1 (2.2)	18 (13.8)	
***Massage therapist*** no yes		38 (82.6)	97 (74.6)	*0.270*
		8 (17.4)	33 (25.4)	
***Aromatherapist*** no yes		44 (95.6)	112 (86.2)	*0.081*
		2 (4.3)	18 (13.8)	
***Osteopath*** no yes		43 (93.5)	113 (86.9)	*0.229*
		3 (6.5)	17 (13.1)	
***Traditional*** no ***Chinese medicine*** yes		45 (97.8)	116 (89.2)	*0.073*
		1 (2.2)	14 (10.8)	
***Yoga teacher*** no		44 (95.6)	114 (87.7)	*0.126*
yes		2 (4.3)	16 (12.3)	
***Practice and products***				
*Conventional Medicine*				
***Prescription only medicine*** no yes		4 (8.7)	14 (10.8)	*0.690*
		42 (91.3)	116 (89.2)	
***Over-the-counter pharmaceuticals*** no yes		11 (23.9)	34 (26.2)	*0.765*
		35 (76.1)	96 (73.8)	
*Complementary Medicine*				
***Aromatherapy oils*** no yes		43 (93.5)	96 (73.8)	*0.005*
		3 (6.5)	34 (26.1)	
***Herbal medicines*** no yes		44 (95.6)	103 (79.2)	*0.010*
		2 (4.3)	27 (20.8)	
***Yoga/tai chi*** no yes		44 (95.6)	102 (78.5)	*0.008*
		2 (4.3)	28 (21.5)	
***Relaxation/meditation*** no		43 (93.5)	91 (70.0)	*0.001*
yes		3 (6.5)	39 (30.0)	
***Vitamin/minerals*** no yes		30 (65.2)	51 (39.2)	*0.002*
		16 (34.8)	79 (60.8)	
***Flower essences*** no yes		45 (97.8)	103 (79.2)	*0.003*
		1 (2.2)	27 (20.8)	

### The association between conventional or complementary health service use

Considering the logistic regression model output presented in Table 4, people with lowered mental health functioning had the highest likelihood of practitioner group consultation with a Western herbalist (OR = 9.17, 95% CI: 1.097–76.84), followed by a counsellor or psychologist (OR = 3.85, 95% CI: 1.488–9.986). They were also more likely to practice relaxation or meditation (OR = 5.10, 95% CI: 1.362–19.129) than use vitamins or minerals (OR = 2.34, 95% CI: 1.061–5.151).

**Table 4:** The association between health service use of people with diabetes mellitus by mental health status

Health Service Use (yes)	Mental Health Status		
	Lowered Functioning ^a^ **(** ***n*** ** = 176)**		
	OR	95% CI	*p*-value
***Practitioners***			
Specialist doctor	0.29	0.125, 0.684	*0.005*
Counsellor/ MH worker	3.85	1.488, 9.986	*0.005*
Western Herbalist	9.18	1.097, 76.84	*0.041*
***Practice or Products***			
Vitamins/Minerals	2.34	1.061, 5.151	*0.035*
Relaxation/meditation	5.10	1.362, 19.129	*0.016*

Note:

a: adjusted for financial situation

### Limitations

This study is limited in its capacity to be generalised to the broader diabetes population due to the cross-sectional study design and the small sample size of people reporting a diabetes diagnosis. In addition, our study employs the MHI-5 which measures quality of life indicators such as psychological distress [7, 38, 39], as well as a predictor for the development of anxiety or depressive disorders [40, 41]. However, the MHI-5 is not specifically focused upon measuring *diabetes-specific distress*, and our results can only provide insights into the influence of lowered mental health functioning in the form of general psychological distress, rather than diabetes-specific distress or anxiety or depression. Despite these limitations, our study findings are strengthened by both the study population being nationally representative of the broader Australian population, and inclusive of both genders, a broad range of ages, as well as our survey design including a comprehensive list of complementary medicine practices, products and practitioner groups.

## Discussion

Our study responds to the call for more research which examines psychological distress and diabetes. Further, research that considers psychological distress and the health service use of people living with diabetes has not been explored. The innovative nature of our study, together with limited related research, has made it difficult to conclusively explain our findings. This has reinforced the need for comprehensive research related to the health behaviours of people living with diabetes and psychological distress, to ensure current diabetes care is meeting the needs of this specific group of people.

Our results indicate that amongst people living with diabetes those with lowered mental health functioning are even more likely to use CM than conventional medicine, when compared to those with normative mental health. Whilst previous research has found people living with diabetes [42, 43] or a mental health concern [34, 44] engage with a variety of CM, a notable finding is that our participants showed a wider choice of CM practices or products, as opposed to CM practitioners. Amongst individuals living with diabetes, our analysis revealed that those with lowered mental health functioning were around three times more likely to consult with a western herbalist than a counsellor or psychologist, when compared to those with normative mental health. Although people with any mental health concern are known to consult with western herbalists [45], it was unexpected that the likelihood of consultation with a counsellor or psychologist was much lower.

A possible explanation is that counsellors/psychologists are not typically trained in CM use [46], which might explain the higher consultation rates with a CM practitioner, such as a western herbalist, for people who choose to include CM as part of their mental health care. Additionally, access to conventional mental health treatment is problematic, including a lack of services and long waiting lists [34, 45]. This might further encourage CM users to seek treatment for mental health concerns with a CM practitioner. Notwithstanding this, our results show a preference for consultation with CM practitioner groups as opposed to conventional practitioner groups. As standard diabetes care teams still consist entirely of conventional medicine practitioners [47, 48, 49], inclusion of CM practitioners as part of diabetes care provision, where appropriate, requires attention.

In terms of CM practice or product use amongst individuals living with diabetes, those with lowered mental health functioning were more than twice as likely to engage in relaxation or meditation than use of vitamins or minerals when compared to those with normative mental health. Relaxation or meditation are often encouraged by both conventional and complementary practitioners for mental health care [50, 51], which could explain this finding. However, vitamin and/or mineral supplementation is also commonly used by people with any mental health concern [52]. However, one theory regarding CM use in the context of a chronic health condition, does help to explain our findings at the broader level.

CM use has been theorised to have different push/pull influences (i.e. push towards CM use/pull away from conventional health care or vice versa) based on a person’s self-perception of the seriousness of their diagnosed chronic condition. People living with diabetes have reported a reluctance to use CM due to the potential for contraindications with commonly prescribed diabetes therapies [53]. An example of this situation, relevant to our CM practice/product findings, is that there is a plethora of over-the-counter vitamin/mineral products available (containing different combinations of vitamins/minerals/herbals) and labelled with words or phrases related to managing psychological distress. Expert knowledge is required as to what specific vitamin or mineral could be helpful in relation to a specific mental health concern, and in what circumstances (e.g. supplementation of b group vitamins for psychological distress as opposed to depression) [52]. This variety of choice, along with varying health literacy amongst CM users in deciding if or which CM product to use, could result in a reluctance for some people living with diabetes to self-prescribe vitamins/minerals as part of their mental health self-care. This may help explain the higher uptake of CM practices, such as relaxation/meditation - which do not contraindicate commonly prescribed diabetes therapies - for those individuals living with diabetes *and* reporting lowered mental health functioning.

Additionally, these same theorised push/pull factors provide further insights regarding the higher likelihood of those living with diabetes and lowered mental health functioning in consulting with a western herbalist. Use of herbal medicines amongst people with any mental health concern is common [34, 45] and our study did identify amongst individuals living with diabetes a significant association and higher rate of herbal medicine use for those with lowered mental health functioning when compared to those with normative mental health. It is possible, at least for people with both diabetes and lowered mental health functioning, that self-prescribing herbal medicine was perceived as too high a risk in light of their diabetes diagnosis, resulting in a desire for professional consultation with a qualified herbalist. Nevertheless, since CM self-prescription of vitamins/minerals or herbals is common and can often go undisclosed to conventional health care providers [54], it is important for those health care practitioners supporting and treating individuals living with diabetes to inquire about CM use, particularly for people with co-morbid mental health.

## Conclusion

People living with diabetes and reporting lowered mental health functioning are using *both* conventional health care and CM, with a preference towards CM use. Research providing detailed examination of the decision-making of people living with diabetes around conventional health care and CM use, particularly in relation to psychological health dimensions such as anxiety, depression or diabetes distress which are common amongst this population, is warranted. Such work will help inform coordinated, effective, safe care which may include use of both CM and conventional health care treatment options (when and where appropriate) for people living with diabetes.

## Data Availability

The datasets used and/or analysed during the current study are available from the School of Public Health, University of Technology Sydney, on reasonable request.
